# Evaluation of a collar‐mounted accelerometer for detecting seizure activity in dogs

**DOI:** 10.1111/jvim.15760

**Published:** 2020-04-15

**Authors:** Karen R. Muñana, Julie A. Nettifee, Emily H. Griffith, Peter J. Early, Nathanael C. Yoder

**Affiliations:** ^1^ Department of Clinical Sciences, College of Veterinary Medicine NC State University Raleigh North Carolina United States; ^2^ Department of Statistics NC State University Raleigh North Carolina United States; ^3^ Whistle Laboratories San Francisco California United States

**Keywords:** activity monitor, canine, clinical trial, idiopathic epilepsy

## Abstract

**Background:**

The majority of dogs with idiopathic epilepsy continue to have seizures despite appropriate treatment.

**Objectives:**

To assess the use of a commercially available, collar‐mounted accelerometer to detect generalized seizures in dogs.

**Animals:**

Twenty two client‐owned dogs with idiopathic epilepsy.

**Methods:**

Six‐month prospective clinical study during which dogs wore a collar‐mounted accelerometer. Seizure documentation was based on owner observations and video recordings. The accelerometer used a predefined algorithm to detect seizures in the first study phase, and an individualized algorithm in the second study phase. Caregivers completed a quality of life (QoL) questionnaire at the initial and final study visit.

**Results:**

Using the predefined algorithm, the accelerometer detected seizures with a sensitivity of 18.6% (95% CI [13.4%, 23.8%]) and mean false detection rate of 0.096/day. Values did not change significantly with use of an individualized algorithm (sensitivity 22.1%, 95% CI [15.1%, 29.0%]; false detection rate 0.054/day). Mean composite QoL score was significantly improved at study completion (50.42) compared to study initiation (39.53; *P* = .005), and this change was moderately correlated with a change in weekly exercise (*r* = 0.46, *P* = .05).

**Conclusions and Clinical Importance:**

Generalized seizures in dogs can be detected with a collar‐mounted accelerometer, but the overall sensitivity is low.

**Abbreviations**
ASD: antiseizure drugs
EEG: electroencephalography
IVETF: International Veterinary Epilepsy Task Force
QoL: quality of life

## 1 INTRODUCTION

Idiopathic epilepsy is a common neurological disorder of dogs that is characterized by recurrent, unprovoked seizures for which an underlying cause cannot be identified other than a confirmed or suspected genetic predisposition,^1^ and for which antiseizure drugs (ASD) remain the cornerstone of treatment. Seizure freedom, the primary goal of treatment, is achieved in as few as 14% of dogs with epilepsy.^2^ For the remainder of the population, the objective of epilepsy management focuses on reducing the seizure frequency to an acceptable level while minimizing medication‐related adverse effects. However, approximately 30% of epileptic dogs never achieve satisfactory seizure control at maximally tolerated doses of 2 or more ASDs,^2^, ^3^ and as such, are considered “drug resistant” based on human guidelines established by the International League Against Epilepsy.^4^

A consistent source of worry for caregivers is the risk and potential consequences of seizures occurring while an epileptic dog is left unattended. This concern is shared by caregivers of humans with epilepsy,^5^, ^6^ and has led to the recent FDA approval of a wrist‐worn, 3‐dimensional accelerometer as a seizure monitoring device in adults with epilepsy (Embrace 2, Empatica Inc, Milan, Italy). Accelerometers are able to reliably detect generalized tonic‐clonic seizures in humans^7^, ^8^, ^9^, ^10^ and the devices have been designed to immediately alert caregivers when a seizure is detected.

Commercially available accelerometers designed for use in dogs have gained popularity as a means to track daily activity and monitor overall fitness and health. Accelerometers have been utilized to objectively measure physical activity and track‐specific movements in dogs involved in studies on behavior,^11^, ^12^ pruritis,^13^, ^14^ osteoarthritis,^15^, ^16^ and weight loss.^17^, ^18^, ^19^ The aim of our study was to evaluate the use of accelerometry to detect seizure activity in dogs. Specific objectives were (1) to determine the accuracy of a commercially available collar‐mounted accelerometer to detect generalized seizures in dogs with idiopathic epilepsy based on a predefined algorithm; (2) to determine whether accuracy could be improved by refining the algorithm based on an individual dog's seizure and activity patterns; and (3) to document any beneficial or adverse effects associated with its use.

## 2 MATERIALS AND METHODS

### 2.1 Study design

The study was conducted at NC State Veterinary Hospital from 2015 to 2018. Inclusion criteria were (1) a diagnosis of idiopathic epilepsy, based on a minimum Tier 1 level of evidence as established by the International Veterinary Epilepsy Task Force (IVETF)^20^ that includes a seizure onset between 6 months and 6 years of age, normal neurological examination, and lack of abnormalities on CBC, chemistry profile, bile acid tolerance and urinalysis to suggest an underlying cause for the seizures; (2) an average of 3 or more generalized seizures per month while being treated with appropriate maintenance ASD administration; and (3) owner access to a smartphone and wireless internet. Owners were required to provide informed consent before study participation and agree to keep their dog confined to a small room or kennel during times the dog was left unattended for the duration of the study. Initial target enrollment of 35 dogs was based on a power analysis performed by a statistician (EHG) using unpublished preliminary data from 5 dogs, and assuming a confidence level of 95%, a hypothesized sensitivity of 90% and a desired level of precision of 10%. Target enrollment was revised to 23 dogs based on an interim analysis performed by the same statistician 6 months before the scheduled study completion date, using data from the first 15 dogs to complete the initial 3‐month phase of the study, to determine whether the results supported an extension of the study duration. The study timeline consisted of 2 phases, each approximately 3 months in duration, with visits scheduled at time 0 (initiation of first phase), 3 months (midpoint, initiation of second phase), and 6 months (study completion). Dogs that met eligibility requirements were provided a collar‐mounted commercially available accelerometer at the first study visit that was to be worn for the duration of the study. Owners were provided study forms on which to record date, time and duration of all observed seizure activity, and to describe the characteristics of the seizure in order to classify it as generalized or focal. To monitor for seizures when dogs were left unattended, owners were provided a battery powered, video surveillance system consisting of 2 easily mounted cameras capable of motion activated and time stamped recordings, cloud storage and data download (Arlo Smart Security System, Arlo Technologies, Inc, San Jose, California). Owners were instructed to keep their dog confined to a small room or kennel when left unattended, and to set up the video surveillance system such that the dog's activity could be monitored in the confined area. Owners were instructed to turn on the video system when leaving the house, and turn it off when returning home. All video data was downloaded at weekly intervals and viewed by study personnel. Any activity suspected of being a seizure was tagged for review and confirmation by 1 of the investigators (KRM) who is a board‐certified veterinary neurologist. Data collection for the first study phase commenced once it was verified that both the accelerometer and video surveillance system were set up correctly. At the midpoint and end of study visits, the accelerometer was inspected and owner records were collected and reviewed. Body weight was recorded at the initial, midpoint, and end of study visits. Owners were required to return the accelerometer and video monitoring system at study completion. The study was approved by the Institutional Animal Care and Use Committee at NC State University.

### 2.2 Accelerometers

To collect movement data during seizures the Whistle Activity Monitor (Whistle Labs Inc., San Francisco, California) was used. The Whistle Activity Monitor is a collar‐mounted, water resistant, 3‐dimensional accelerometer that uses a wireless internet connection to upload data and algorithms to identify different activities. The device has a battery life of 10 days, an 8‐bit resolution and samples data at 100 Hz. The data is automatically uploaded to Whistle's servers on a periodic basis when the device is around a wireless or Bluetooth device with a connection to the internet. The Whistle Activity Monitor is the first‐generation device produced by Whistle Labs that was commercially available between 2013 and 2016, and has since been replaced by a newer model.

### 2.3 Seizure detection

To create a preliminary seizure detection algorithm, a pilot study was previously conducted to capture seizure related movements in epileptic dogs wearing the activity monitor. Seizure activity was recorded manually by owners, and accelerometer data were manually reviewed. The review and data cleansing procedure yielded 19 potential seizures from 2 dogs. To aid in distinguishing the characteristics of the measurements during a seizure and differentiate these from other activities, the sensor's measurements were transformed into the dog's coordinate system using the physical constraints of a collar‐mounted device and assumptions about the dog's posture. Utilizing these data, an algorithm was developed using a machine learning based approach that has resulted in improvements in the accuracy of wrist worn accelerometers used for seizure detection in humans.^21^ For our study, a random forest classifier was used as it has shown promise in some human activity recognition work.^22^ The input features used for the random forest model included both time and frequency domain features calculated over a 4‐second window that was turned into a single prediction of the probability of a seizure occurring during that time. The frequency domain features were the average log‐spectra and the spectral‐entropy of the signal. The time domain features were the value of the 5th, 10th, 25th, 50th, 75th, 90th, and 95th quantiles of each axis of the oriented accelerometer data. In order to reduce false positive readings, the output of the algorithm was smoothed using a 5‐point median filter and when the filtered value was above 0.5, a seizure was predicted as having occurred. For the individualized algorithm used in the second phase of the study, the same random forest classifier was used but the threshold value was adjusted based on the phase 1 results. The threshold was manually adjusted by raising the minimum probability required for a seizure to be classified to slightly below the minimum value of a true positive for dogs that had numerous false positive detections during phase 1, and lowering the threshold if the dog had many false negative detections.

### 2.4 Quality of life (QoL) questionnaire

To assess for beneficial or adverse effects associated with the use of the activity monitors, owners were asked to complete a QoL survey at study initiation and again at study completion (Data S1). The survey was adapted from a questionnaire validated for use in dogs with epilepsy^23^ and designed to assess the QoL of both the dog and the caregiver. The survey consisted of 15 questions with a bipolar response scale of 1 (agree strongly) to 5 (disagree strongly). Questions were worded such that a better QoL corresponded to a response of 1 in some instances and a response of 5 in others. For the analyses, scales from all questions were standardized such that a high response to any question was positive. A composite QoL score was calculated by compiling responses to the questionnaire with a possible score of between 15 and 75, and a higher overall score indicating a better QoL. The QoL survey also included open ended questions in which owners were asked to estimate the amount of time dogs spent in different forms of exercise each week.

2.5

For each dog, the date and time of all generalized seizures was compiled from owner competed study forms and video recordings. This was performed by an investigator (KRM) who was blinded to the data acquired by the accelerometer. Similarly, accelerometer activity that met the algorithm threshold for a seizure was compiled by an investigator (NCY) blinded to the dogs' actual seizure activity. A record was also created of time periods during which the accelerometer was not collecting data because it was being charged or was not being worn by the dog for another reason. The sensitivity of the device was determined by calculating the ratio of the number of seizures that the accelerometer correctly detected to the total number of seizures. Any seizure that occurred during a period of time when the accelerometer was not collecting data was not used in the calculations. The false detection rate was calculated as the number of false detections in a study phase divided by the number of days in the study phase. One day was subtracted from the number of days in the study phase for any 24‐hour time period during which the accelerometer was not collecting data, and this corrected value was used to calculate the false detection rate.

### 2.6 Statistical analysis

Summary statistics for normally distributed continuous variables are reported as mean, SD, and range, and data not normally distributed are reported as median and range. Composite QoL score and weekly exercise (minutes) were compared between study initiation and study completion, and body weight was compared at study initiation, midpoint and study completion using a paired *t* test. Device sensitivity was analyzed using a logistic regression model with a fixed period effect for study phase, a random subject effect and covariates of weight, weekly exercise (minutes), and composite QoL scores. The false detection rate per day was modeled using a standard mixed effects linear model with a fixed period effect for study phase, a random subject effect, and covariates of weight, weekly exercise (minutes), and composite QoL scores. To evaluate for any change in seizure frequency during the study that might have influenced results, a standard mixed effects linear model was used including the same parameters as that for false detection rate. Residuals were evaluated for heteroscedasticity, in which case, a natural log transformation was performed on the rate to determine whether the resulting model fit was improved. Associations between changes in QoL and seizure frequency, body weight and weekly exercise (minutes) were evaluated using a Pearson's correlation coefficient. Analyses were performed using commercially available statistical software (SAS version 9.4, Cary, North Carolina) with a significance level of *P* < .05.

## 3 RESULTS

**FIGURE 1:**
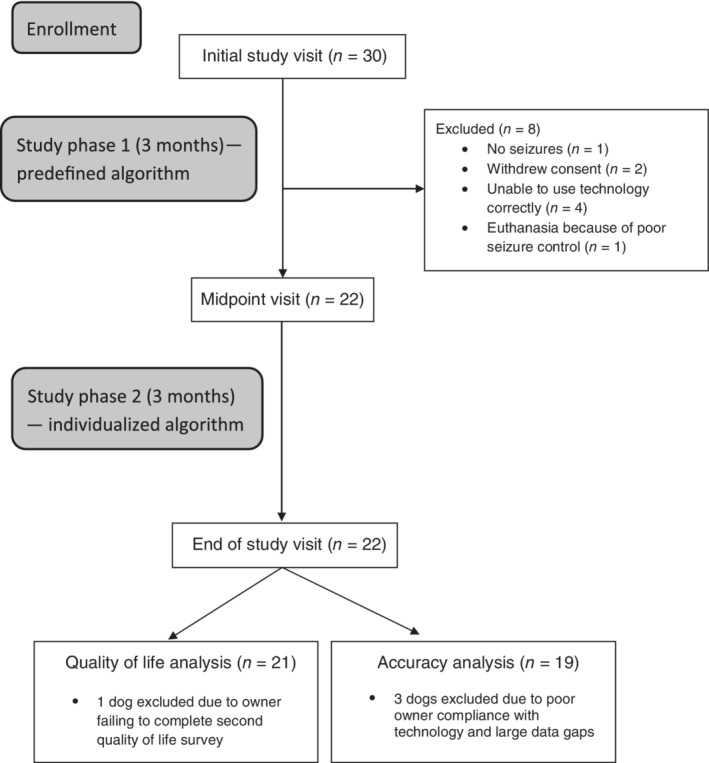
Disposition of dogs participating in the study

Thirty dogs were enrolled in the study (Figure 1). Eight dogs were excluded from the study during the first phase, for the following reasons: lack of seizures (n = 1), euthanasia because of poor seizure control (n = 1), owner withdrew consent (n = 2), and owner unable to comply with correct use of the technology (n = 4). All 22 dogs that completed the first study phase continued on to study completion. Review of all data at the completion of the study identified 3 dogs with substantial gaps in the data collected from either the accelerometer (n = 2) or the video monitoring system (n = 1), and these dogs were excluded from the accuracy analysis.

Of the dogs to complete the study, breeds represented included mixed breed (n = 8), Shetland sheepdog (n = 2), German shepherd dog (n = 2), and 1 each of Australian shepherd, Beagle, Boxer, English bulldog, German wirehaired pointer, Golden retriever, Irish wolfhound, Labrador retriever, Viszla, and Wheaton terrier. There were 8 spayed females and 14 neutered males. The mean age at study enrollment was 4.5 years (SD, 1.5 years; range 1‐7 years), with a mean duration of epilepsy of 2.3 years (SD, 1.4 years; range 0.5‐6 years). Thirteen dogs received a diagnosis of idiopathic epilepsy based on an IVETF Tier 1 level of confidence; all of these dogs had a CBC, serum biochemistry profile, bile acid tolerance and urinalysis performed. The remaining 9 dogs underwent additional neurodiagnostic testing including brain imaging (magnetic resonance imaging in 8 dogs, computed tomography in 1 dog) and cerebrospinal fluid analysis, leading to a diagnosis of idiopathic epilepsy with an IVETF Tier II level of confidence. All study dogs were being treated with ASDs. Two dogs were being administered 1 ASD, 6 dogs were being administered 2 drugs, 12 dogs were on 3 ASDs, and 2 dogs were on 4 ASDS. Antiseizure drugs being utilized included phenobarbital (n = 14), potassium bromide (n = 8), zonisamide (n = 17), immediate release levetiracetam (n = 6), extended release levetiracetam (n = 9), gabapentin (n = 3), lorazepam (n = 1), and chlorazepate (n = 1).

A total of 215 generalized seizures were documented in the study population during the first study phase, with a median of 7 seizures per dog (range, 1‐40) and a mean seizure frequency of 0.11/day (SD, 0.11; range, 0.0065‐0.41/day). Of these 215 seizures, 40 were detected by the device using the predefined algorithm, for an overall sensitivity of 18.6% (95% CI [13.4%, 23.8%]). Sensitivity in individual dogs ranged from 0% to 78% with a mean of 24.9% (95% CI [12.4%, 37.4%]). There were 184 false detections in the population of dogs during the first study phase, with a mean false detection rate per dog of 0.096/day (SD, 0.12; range, 0‐0.36/day).

**FIGURE 2:**
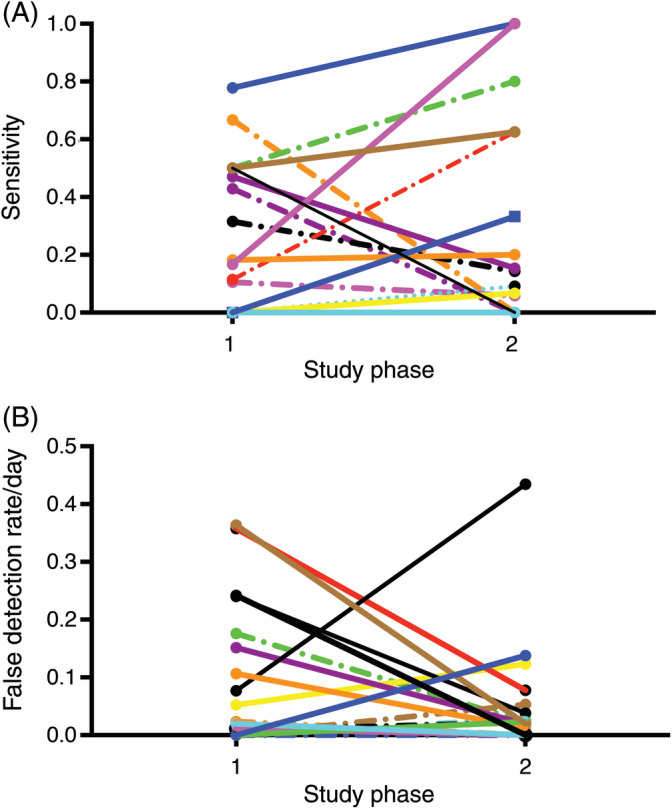
Performance of the accelerometer in detecting seizures in study dogs using a predefined algorithm (study phase 1) and an individualized algorithm (study phase 2). Sensitivity is depicted in A, and false detection rate in B. Each colored line represents an individual dog

During the second study phase, a total of 136 generalized seizures were reported, with a median of 5 seizures per dog (range, 1‐17) and a mean seizure frequency of 0.073/day (SD, 0.06; range, 0.0068‐0.21/day). Thirty of these generalized seizures were detected by the device using the individualized algorithm, yielding an overall sensitivity of 22.1% (95% CI [15.1%, 29.0%]). Sensitivity in individual dogs ranged from 0 to 100%, with a mean of 26.8% (95% CI [9.7%, 43.9%]). There were 96 false detections in the population of dogs during the second study phase, with a mean false detection rate per dog of 0.054/day (SD, 0.10; range, 0‐0.43). With use of an individualized algorithm, seizures were detected with a 100% accuracy in 2 dogs (sensitivity 100%, false detection rate 0/day). Device sensitivity and false detection rate for the population of dogs is summarized in Figure 2. Detail on the performance in individual dogs is provided as Table S1. There was no statistically significant difference in seizure frequency (*P* = .23), device sensitivity (*P* = .82), or false detection rate (*P* = .24) between study phases.

**FIGURE 3:**
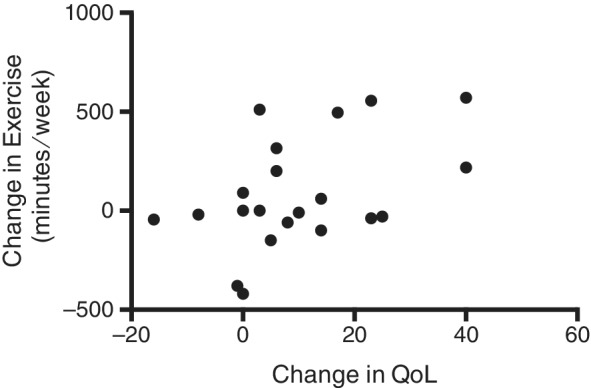
Scatter plot of the correlation between change in quality of life (QoL) and change in weekly exercise between the end of study and study initiation. *r* = 0.46, *P* = .05

No adverse effects were reported with use of the accelerometer. However, 1 dog that regularly swam in a backyard pool was withdrawn from the study because of repeated malfunction of the device associated with the water immersion. No significant difference was demonstrated in weekly exercise between study initiation and study completion (269 versus 369 minutes, respectively; SE, 65.83, *P* = .15). Similarly, the mean body weight at study initiation (28.45 kg) did not differ significantly from mean body weight at study midpoint (29.18 kg; SE 0.77, *P* = .38) or at study completion (29.79 kg; SE, 0.88, *P* = .15). Mean composite QoL score at the end of the study (50.42) was significantly increased compared to study initiation (39.53; SE, 3.37; *P* = .005). No correlation was demonstrated between the changes in QoL and seizure frequency or body weight, but a moderate positive correlation was noted between change in QoL and change in weekly exercise (*r* = 0.46, *P* = .05; Figure 3).

## 4 DISCUSSION

Our study evaluated the clinical reliability of a commercially available, collar‐mounted accelerometer to detect generalized seizures in dogs in the home setting. We demonstrated that seizures in dogs can be detected with accelerometry but with low sensitivity with the algorithms used. The predefined algorithm accurately detected approximately 20% of the seizures, and this did not improve significantly when the threshold used to determine when a seizure occurred was adjusted based on the dog's previously reported seizures. The device performed better with respect to its ability to discriminate activities that were not seizures, with the number of false detections per dog averaging between 0.054 and 0.096/day. Although our study did not involve a therapeutic intervention, owners reported a significant improvement in QoL during the course of the study.

Wrist‐worn accelerometers have become a valuable tool in the management of epilepsy in humans. Initial validation of the technology was performed in hospitalized patients admitted to long‐term video‐electroencephalography (EEG) monitoring units, during which all seizure activity was confirmed by EEG and all movements recorded, with reported sensitivity ranging from 85 to 100% and a false detection rate of 0.2‐1/day.^8^, ^9^, ^24^ A subsequent field study designed to evaluate the use of the device in the home environment reported a mean sensitivity of 85% and a mean false detection rate of 1.4/day.^7^ A major objective for use of these devices in humans is the detection of severe nocturnal seizures, in order to enable intervention and lead to the prevention of sudden unexpected death in epilepsy. A survey designed to assess opinions regarding the use of seizure detection devices among epileptic patients, caregivers and doctors demonstrated that the majority of respondents required at least a 90% sensitivity for a device to be considered useful.^25^ The acceptable rate of false alarms among respondents was, on average, 1 false alarm per seizure (calculated by dividing the number of false alarms allowed per week by the weekly seizure frequency), or 1 false alarm per week in seizure‐free patients.^25^ Hence, in humans, optimizing sensitivity assumes the greatest importance. In contrast, as part of the algorithm development process for our study, a focus was placed on minimizing the number of false positive detections. This was based on the premise that the device would be most useful to owners when their dog is left unattended during the day, and should be designed to avoid incorrectly alerting the owner. It is likely that a better sensitivity could have been achieved at the expense of a higher false positive rate. As it is, the daily false positive detection rates seen during our study were over an order of magnitude lower than those reported with the use of wrist‐worn devices in the home environment.^7^ Acceptable values for sensitivity and false detection rates for a device to be used to detect seizures in dogs have not been established.

The ability of accelerometry to detect seizures depends on the seizure type, with the highest sensitivity in humans reported for generalized tonic‐clonic seizures.^26^ Furthermore, the physical manifestations of the seizure have been shown to influence the accuracy, independent of seizure type. For a wrist‐worn accelerometer, seizures that manifest with rhythmic, shaking arm movements are more likely to be detected than seizures without vigorous movements of the arm.^26^ The lower sensitivity of the accelerometer in detecting seizures in dogs compared to its use in humans is presumed to be due in part to the position of the accelerometer on the body. It seems likely that a device worn on the neck, as in our study in dogs, would need to be more sensitive in detecting seizure activity compared to a wrist‐worn unit in humans, based solely on the intensity of rhythmic activity of the limbs compared to the neck in most tonic‐clonic seizures. While designing the study, the investigators considered attaching the accelerometer to the limb of a dog, but could not devise a method that was secure, and in a location that would not enable the device to be chewed and protected from damage during movement. The collar‐mounted device was opted for its ability to easily and securely be attached to the dog in a relative protected location.

There was a large degree of variability in the accuracy of the accelerometer to detect seizures among study dogs. Using the individualized algorithm in the second study phase, the sensitivity ranged from 0% to 100% and the false detection rate varied from 0 to 0.43/day. The device was shown to have an accuracy of 100% in 2 study dogs during the second study phase, with 100% sensitivity and no false detections, and these dogs were not part of the pilot study that was used to develop the initial algorithm. However, there were only a total of 3 reported seizures for these 2 dogs during the second study phase. The variability in accuracy is believed to be because of the differences in the dogs' normal activity patterns as well as differences in the physical manifestations of the seizure. Although all dogs in the study had generalized seizures, differences in the characteristic movements during a seizure are likely to influence the ability for the seizure to be detected by accelerometry. To further evaluate this variability and the characteristic movements that are more likely to be detected, video recordings of seizure activity for all the study dogs would be required. However, the video monitoring system was only employed when caregivers were not at home with their dog, and therefore video recordings of seizure events were not collected from many of the study dogs. Video recordings would similarly be required to discern activities that induce false detections in dogs, and without such information the cause of the false detections identified in our study cannot be further characterized. False alarms in humans are attributed to specific repetitive activity patterns such as tooth brushing, playing games, hand shaking, dancing, and clapping.^7^ Another factor that may have also contributed to the dog‐to‐dog variability is the limited size of the training set. Because seizures from only 2 dogs were used to generate the classifier, individual dogs could not be held out of the cross‐validation process during model creation which may have resulted in a model that was overfit to the characteristic seizure movements of these 2 dogs during the pilot study. This small sample training set likely greatly hampered the ability of the algorithm to generalize to other dogs and only adjusting the probability model in order to individualize the model was likely unable to compensate for this fact.

The use of machine learning to further refine algorithms has resulted in improvements in the accuracy of wrist‐worn accelerometers used for seizure detection in humans.^21^ Furthermore, evaluating an established algorithm on a large data set can lead to improvements in accuracy.^27^ Much of the recent progress in machine learning and activity recognition in general has been because of the application of methodologies such as deep learning that greatly benefit from large amounts of training data, and obtaining satisfactory performance with small amounts of training data is an area of active research. In our study, utilizing a machine learning model that was generated using only pilot data from 2 dogs limited the overall sensitivity and robustness of the model, and developing a model using a double cross‐validation approach such as that used in humans^27^ remains future work. It is expected that the reliability of the collar‐mounted accelerometer in dogs to detect seizures could be improved further with additional data collection and algorithmic refinements.

A significant improvement in caregiver reported QoL was noted over the course of the study. This was moderately associated with an increase in time spent exercising each week, but was independent of any change in seizure frequency or body weight. Weekly exercise and body weight were assessed in our study to determine if use of the accelerometer was associated with any changes in activity level or body condition, and neither of these parameters were noted to change significantly. Accelerometers have been shown to have some positive effect on the time spent engaging in physical activity in humans,^28^ and the degree of physical activity has been associated with improvement in health related QoL.^29^ The accelerometer used for our study provides real time data on dog's activity to the owner via a smartphone, and allows the owner to set goals for the dog, similar to commercially available activity trackers for humans. An owner may engage in more physical activity with the dog when the accelerometer is being used, particularly if goals have been established, and it seems feasible that that this increase in physical activity might be associated with some increase in QoL.

However, because the QoL questionnaire addresses issues specific to the management of epilepsy, it seems unlikely that the noted improvement in QoL is because of an increase in exercise alone. The improvement could also reflect caregivers worrying less about leaving their dog unattended during the study. The video recording system allowed dogs to be viewed in real time from a remote location, and several of the dog owners reported using this feature. The emotional burden associated with caring for an epileptic dog is well described. In a long‐term epidemiological study of idiopathic epilepsy, 60% of caregivers reported a negative influence on their daily life.^30^ The most common complaints expressed by caregivers included feelings of restriction in lifestyle, stress that the dog might have a seizure while unattended, and fear of coming home and finding the dog dead after a seizure. In a separate study designed to explore caregivers' perspectives on managing a dog with idiopathic epilepsy, 36% of respondents indicated that their dogs' seizures affected their ability to stay away overnight because of worry over leaving their dog.^31^ Any intervention that might alleviate caregivers worry could be expected to increase QoL. Finally, it is also possible that the improved QoL is because of study participation. The Hawthorne effect is a well described phenomenon in which individuals experience a non‐specific beneficial effect from participating in clinical trials.^32^ This effect has been attributed to the attention participants receive from investigators during the course of the study, which often is rewarding on its own, and can influence participants' response to health‐related QoL questionnaires. An improvement in QoL in the absence of a positive treatment effect was reported in a clinical trial evaluating levetiracetam as add‐on treatment for dogs with epilepsy, and the Hawthorne effect was cited as a possible explanation.^33^ The improvement in QoL identified in our study is presumed to be because of a combination of these factors, but the relative contribution of increased exercise, video monitoring and study participation is unknown.

The main limitation of our study is the lack of EEG confirmation of seizure activity and the dependence on owners for the seizure data used in the analyses. Ideally, data on seizures and movements would be collected with the use of continuous video‐EEG monitoring, as has been performed in human studies. However, this would prove technically challenging in dogs for several reasons, including the need to maintain EEG recordings for an extended period of time, the inability to capture data on normal activities in a dog confined to a small space that allows for video recording, and the potential for a lengthy hospitalization to acquire data on multiple seizures. Instead, the study required owners to keep a record of all witnessed seizure activity, and these records are subject to bias. Because seizure detection was not based on EEG confirmation as is the gold standard, it is possible that actual seizures were not detected by the accelerometer. It is also possible that events classified as false detections may have been actual seizures, although the inclusion of only generalized seizures in the study makes this less likely.

In addition, data capture from both the accelerometer and video recording system relied on owners' proper use of the equipment, including maintaining the charge on the accelerometer, keeping the accelerometer attached to the dogs' collar at all times except during charging, setting up the video monitoring system such that dogs could be viewed when left unattended, and turning on the video monitoring system each time the owners left the house. There were expected compliance issues with the equipment, leading to gaps in the data for some of the dogs. This was accounted for by careful review of the data, and exclusion of any seizures that occurred during a time period when the accelerometer was not operational. It was more difficult to determine whether the video monitoring system was used at all times when the dog was left unattended, and this could have biased the data. Three study participants were excluded from the accuracy analysis because of substantial known gaps in the data collected from the activity monitor or video monitoring system. The requirement that dogs be confined to a small area when left unattended to allow for video monitoring is another study limitation. It is possible that the activities captured by the accelerometer during these times are not reflective of the dog's movements in a more natural home setting, and differences in level or type of movements could potentially influence the false detection rate. The inclusion of a single investigator to determine whether a seizure was observed on video recordings is a potential weakness of the study design. The study focused on generalized motor seizures, and as such, the identification of a seizure is relatively straightforward. Nonetheless, the reliability of this determination may have been improved by utilizing more than 1 reviewer.

Finally, the study did not meet the target enrollment of 23 dogs. Study enrollment was terminated after the target of 23 dogs was met, but 1 of the later dogs to enroll withdrew in the first study phase and an additional 3 dogs were excluded after study completion because of large gaps in the data associated with poor compliance with the equipment. Consequently, data from only 19 dogs were available for the accuracy analyses. Based on the low sensitivity that was demonstrated in the first study phase, it was decided to not further extend the study duration to enroll additional dogs. The study was not able to demonstrate that an individualized algorithm resulted in a significant improvement in the device's accuracy in detecting seizures This may be because of the small study population as well as the low number of seizures that were captured on video, both of which limited the available data from which the algorithm could be improved.

To be included in the study, dogs were required to have an average of 3 or more generalized seizures per month based on owner diaries. This criterion was established to ensure a sufficient number of seizures to analyze. Once enrolled in the study, a specific seizure frequency was not required for continued participation, although dogs that did not have any seizures during the first 3 months of the study were disqualified. A frequency of 3 or more seizures a month was reported in only 8 dogs in the first study phase and 6 dogs in the second study phase. This decrease in seizure frequency from the time of study inclusion may be because of regression to the mean, a statistical term used to describe the fluctuation of biological variables over time, that has been proposed to play a role in the placebo response demonstrated in epilepsy trials in dogs.^34^ Owners may be more likely to participate in a clinical trial when their dog's seizures are not well controlled, and improvement in seizure frequency is probable over the short term because of time alone. In addition, because this was not a therapeutic trial, treatment changes were not prohibited during the study, and it is possible that changes to the ASD regimen resulted in improved seizure control. Regardless of the cause, the lower than expected seizure frequency among study participants prevented the acquisition of additional seizure data that might have resulted in an improvement in the device algorithm.

## OFF‐LABEL ANTIMICROBIAL DECLARATION

Authors declare no off‐label use of antimicrobials.

## CONFLICT OF INTEREST DECLARATION

Nathanael Yoder is employed by Whistle Labs, the company that manufactured and sold the accelerometer evaluated in our study.

## INSTITUTIONAL ANIMAL CARE AND USE COMMITTEE (IACUC) OR OTHER APPROVAL DECLARATION

Approved by the NC State University IACUC (Protocols 15‐053‐O and 18‐013‐O).

## HUMAN ETHICS APPROVAL DECLARATION

Authors declare human ethics approval was not needed for our study.

## Supporting information


**Table S1.** Sensitivity and false detection rates for individual dogs in the study.

**Data S1.** QoL questionnaire used in the study.
