# Supplementary material for: Evaluation of a collar‐mounted accelerometer for detecting seizure activity in dogs
Source: J Vet Intern Med. 2020 Apr 15;34(3):1239–47. doi: 10.1111/jvim.15760 (PMC7255659; doi:10.1111/jvim.15760)
Supplement: Supplementary file 1 — Table S1. Sensitivity and false detection rates for individual dogs in the study. [file JVIM-34-1239-s001.pdf]

Supplementary Table 1: Sensitivity and false detection rates for individual dogs in the study

	**Period 1 – Predefined Algorithm**				**Period 2 – Individualized Algorithm**			
**Dog**	**Seizures**	**Seizures Detected**	**Sensitivity**	**False Detection Rate/Day**	**Seizures**	**Seizures Detected**	**Sensitivity**	**False Detection Rate/Day**
1	6	3	0.5	0.24	4	0	0	0
2	40	0	0	0	3	1	0.33	0.14
3	2	1	0.5	0.36	8	5	0.63	0.02
4	1	0	0	0.02	4	0	0	0
5	6	0	0	0	6	0	0	0.023
6	6	1	0.17	0.011	1	1	1	0
7	11	2	0.18	0.24	5	1	0.2	0.038
8	17	8	0.47	0.11	13	2	0.15	0.012
9	35	4	0.11	0.077	16	10	0.63	0.43
10	6	0	0	0.15	15	1	0.067	0.019
11	19	6	0.32	0.36	7	1	0.14	0.078
12	4	0	0	0.052	3	0	0	0.12
13	14	0	0	0.0089	12	0	0	0.021
14	9	7	0.78	0	2	2	1	0
15	8	0	0	0	11	1	0.091	0.053
16	2	1	0.5	0	5	4	0.8	0.029
17	19	2	0.11	0.18	17	1	0.059	0.022
18	3	2	0.67	0	3	0	0	0.028
19	7	3	0.43	0.024	1	0	0	0
