# Supplementary material for: Evaluation of a collar‐mounted accelerometer for detecting seizure activity in dogs
Source: J Vet Intern Med. 2020 Apr 15;34(3):1239–47. doi: 10.1111/jvim.15760 (PMC7255659; doi:10.1111/jvim.15760)
Supplement: Supplementary file 2 — Data S1. QoL questionnaire used in the study. [file JVIM-34-1239-s002.pdf]

QUALITY OF LIFE QUESTIONNAIRE

Name: ____ Date: ______________

For each of the following, circle the most appropriate answer:

		**Agree Strongly**	**Agree**	**Neutral**	**Disagree**	**Disagree Strongly**
1.	In the last 3 months, the frequency of seizures in my dog was acceptable.	1	2	3	4	5
2.	In the last 3 months, the severity of seizures in my dog was acceptable.	1	2	3	4	5
3.	In the last 3 months, the seizures in my dog were well managed overall.	1	2	3	4	5
4.	In the past 3 months, my dog's attitude has been good.	1	2	3	4	5
5.	In the past 3 months, my dog's activity level has been good.	1	2	3	4	5
6.	In the past 3 months, my dog's overall quality of life has been good.	1	2	3	4	5
7.	In the past 3 months, I worried about the frequency of seizures in my dog.	1	2	3	4	5
8.	In the past 3 months, I worried about the severity of seizures in my dog.	1	2	3	4	5
9.	I experience limitations in my work, education, or day to day activities because of my dog's seizures.	1	2	3	4	5
10.	I experience limitations to my social life because of my dog's seizures.	1	2	3	4	5
11.	Overall, caring for my epileptic dog causes limitations on my life.	1	2	3	4	5

Questions continue on other side.

		**Never**	**Rarely**	**Some-times**	**Often**	**Very Often**
12.	In the past 3 months, how often did you feel like your dog's epilepsy caused conflict with your work, education, or day to day activities?	1	2	3	4	5
13.	In the past 3 months, how often did you feel that your dog's epilepsy limited your social life?	1	2	3	4	5
14.	In the past 3 months, how often did your feel that your dog's epilepsy limited your independence?	1	2	3	4	5
15.	In the past 3 months, how often has your dog had structured exercise (walks, play, etc.) each day?	1	2	3	4	5

From the following list, please choose the exercise that your dog has engaged in over the last 3 months, and estimate the time engaged in these activities per week.

Activity — Estimated Amount of Time Per Week

☐ Walk __________minutes

☐ Run __________minutes

☐ Play with other dog __________minutes

☐ Fetch __________minutes

☐ Hike __________minutes

☐ Agility (or similar training/event) __________minutes

☐ Other (specify) ______________ __________minutes
